# Molecular characterization of *Clostridium perfringens* isolates from a tertiary children’s hospital in Guangzhou, China, establishing an association between bacterial colonization and food allergies in infants

**DOI:** 10.1186/s13099-023-00572-x

**Published:** 2023-10-08

**Authors:** Kun-yi Huang, Bing-shao Liang, Xiao-yan Zhang, Huan Chen, Ni Ma, Jiao-li Lan, Ding-You Li, Zhen-wen Zhou, Min Yang

**Affiliations:** 1 Department of Gastroenterology, Guangzhou Women and Children’s Medical Center, Guangzhou Medical University, Guangzhou, China; 2 Guangdong Provincial Clinical Research Center for Child Health, Guangzhou Women and Children’s Medical Center, Guangzhou Medical University, Guangzhou, China; 3 Department of Pediatrics, Guangdong Provincial People’s Hospital (Guangdong Academy of Medical Sciences), Southern Medical University, Guangzhou, China; 4 Division of Gastroenterology, Children’s Mercy Hospital, University of Missouri Kansas City School of Medicine, Kansas City, USA; 5 Clinical Laboratory, Longgang Maternity and Child Institute of Shantou University Medical College (Longgang District Maternity & Child Healthcare Hospital of Shenzhen City), Shenzhen, China; 6 Department of Pediatrics, The Third Affiliated Hospital of Sun Yat-sen University, Guangzhou, China

**Keywords:** *Clostridium perfringens*, Food allergy, Cow's milk protein allergy, Infants

## Abstract

**Background:**

Cow’s milk protein allergy (CMPA) is one of the most common types of food allergy in infants. Faecal pathogen cultures showed that the positive rate of Clostridium perfringens was more than 30%, which was significantly higher than that for other bacteria. Therefore, it is speculated that Clostridium perfringens colonization may be one of the pathogenetic factors for CMPA in infants. We conducted a real-world evidence study. Infants aged 0–6 months with diarrhoea and mucoid and/or bloody stools were recruited from a large tertiary hospital in China. Faecal pathogen cultures for the detection of Clostridium perfringens were confirmed by flight mass spectrometry, and potential toxin genes were identified using PCR. After 12 months of follow-up, the diagnoses of CMPA and food allergy were recorded. The correlation was assessed by Pearson correlation analysis.

**Results:**

In this study, 358 infants aged 0–6 months with gastrointestinal symptoms and faecal pathogen cultures were recruited. A total of 270 (44.07% girls; mean age, 2.78 ± 2.84 months) infants were followed up for 12 months. Overall, the rate of positivity for *Clostridium perfringens* in faecal pathogen cultures was 35.75% (128/358) in infants aged ≤ 6 months. The earliest *Clostridium perfringens* colonization was detected within 2 days after birth. The majority of *Clostridium perfringens* isolates were classified as type C in 85 stool samples. In the *Clostridium perfringens*-positive group, 48.21% (54/112) of infants were clinically diagnosed with food allergies after 12 months, including 37.5% (42/112) with CMPA, which was significantly higher than that of the negative group, with 7.59% (12/158) exhibiting food allergies and 5.06% (8/158) presenting CMPA (P < 0.0001). Faecal *Clostridium perfringens* positivity was significantly correlated with CMPA, food allergy, faecal occult blood, faecal white blood cells, antibiotic use, increased peripheral blood platelet counts, and decreased haemoglobin levels (P < 0.0001).

**Conclusions:**

This study demonstrates that intestinal colonization by *Clostridium perfringens* is common in infants. The majority of *Clostridium perfringens* isolates are classified as type C. Colonization of the intestine by *Clostridium perfringens* is associated with the development of CMPA and food allergy in infants.

**Supplementary Information:**

The online version contains supplementary material available at 10.1186/s13099-023-00572-x.

## Background

 From early life, the intestinal flora plays a crucial role in shaping the immune system, preventing colonization by pathogenic microorganisms, maintaining intestinal homeostasis, and improving the health and development of infants. The microbiota alters the structure and function of the immune system and reshapes the immune microenvironment. Disordered gut microbiome development could aggravate balanced microbiome-host interactions and promote or interfere with the development of specific diseases, including food allergic diseases (atopy and asthma) and gastrointestinal disorders (diarrhoea, inflammatory bowel disease, and necrotizing enterocolitis) [1–4].

*Clostridium perfringens* is a gram-positive, nonmotile, anaerobic bacillus widely present in the gastrointestinal tract of healthy humans and animals. Studies demonstrated intestinal colonization by *Clostridium perfringens* in 27.2% of faeces from infants ≤ 1 year of age in Jordan, and infants aged ≤ 6 months had a higher colonization rate than older infants [5]. A similar study from Japan found *Clostridium perfringens* in the faeces of 18.2% of healthy infants who were approximately 30 days old [6]. The rate of colonization by *Clostridium perfringens* in caesarean-born infants is higher than that in vaginally born infants and can be affected by the type of feeding during the first month of birth [7].

*Clostridium perfringens* and *Clostridium difficile* are pathogenic clostridia potentially associated with gastrointestinal infections and allergies in infants [8]. *Clostridium perfringens* colonization in neonates is associated with necrotizing enterocolitis (NEC) development and severity [9]. However, the relationship between intestinal colonization of Clostridium perfringens and food allergies or cow’s milk protein allergy (CMPA) has not been reported. We hypothesize that intestinal colonization of *Clostridium perfringens* in infants is associated with food allergy development.

## Results

### Clostridium perfringens colonization and clinical features

**Fig. 1:**
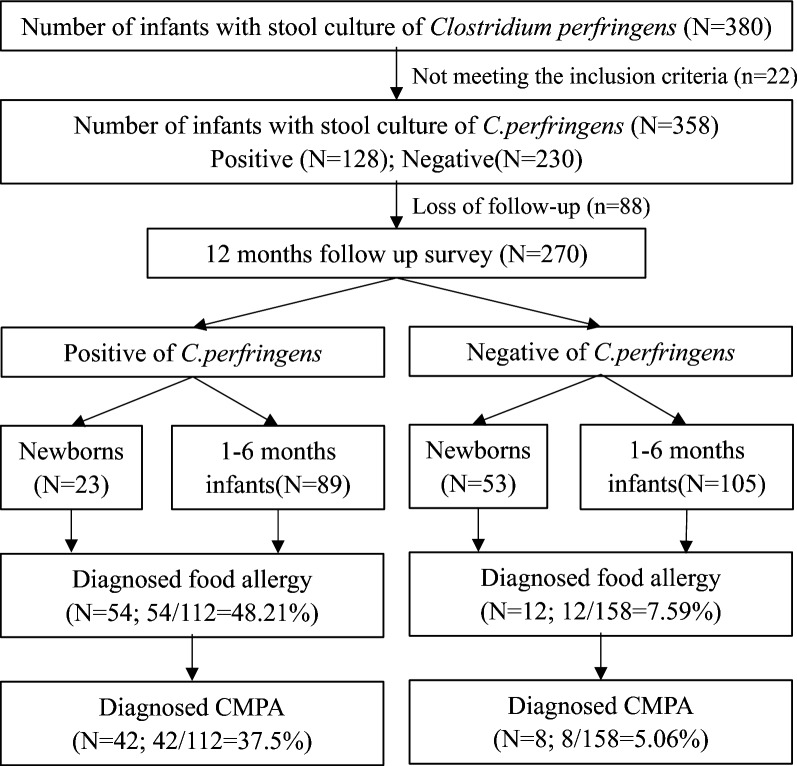
Flow diagram of the patient group selection and outcome

As shown in Fig. 1, a total of 358 infants aged 1 day to 6 months had stool samples sent for pathogen culture, and 128 of these children were positive for *Clostridium perfringens.* The earliest gut *Clostridium perfringens* colonization was detected within 2 days after birth. Overall, the *Clostridium perfringens* colonization rate was 35.75% (128/358). A total of 270 infants (44.07% girls; mean age, 2.78 ± 2.84 months) were enrolled in a 12-month follow-up survey (Table 1). There were no significant differences in sex or age between the *Clostridium perfringens-*negative and *Clostridium perfringens*-positive groups (*P* > 0.05), whereas there were significant differences in disease duration (*P* < 0.001). The rates of positivity for *Clostridium perfringens* colonization were 32.14%, 46.43%, and 21.43% in the groups exclusively fed with mother’s milk, fed with mother’s milk supplemented with formula, and exclusively fed with formula, respectively (*P* < 0.05) (Table 1).

The main presenting symptoms were bloating, diarrhoea and mucoid and/or bloody stools. The incidence of bloating and mucoid and/or bloody stools in infants with or without *Clostridium perfringens* colonization significantly differed (P < 0.05) (Table 1). The main clinical diagnoses included NEC, FPIAP, colitis and diarrhoea. Compared with those in the *Clostridium perfringens-*negative group, the rates of NEC and FPIAP in the positive group were significantly increased (Table 1). Moreover, the rate of metronidazole administration in the *Clostridium perfringens-*positive group was higher than that in the *Clostridium perfringens*-negative group (33.9% vs. 10.76%) (*P* < 0.0001). A total of 231 patients underwent routine blood tests. A total of 27.27% (63/231) of patients had elevated white blood cell counts (> 12.0 × 10^9^/L, range 12.2 to 18.6 × 10^9^/L), 18.18% (42/231) of patients had elevated platelet counts (> 400 × 10^9^/L), and 13.4% (31/231) of patients had decreased neutrophil counts (< 1.5 × 10^9^/L). A total of 42.42% (98/231) of patients had anaemia (HB < 120 g/L), including 6 patients (2.59%) with moderate anaemia (HB < 90 g/L). There were significant differences in the number of leucocytosis, thrombocytosis and neutropenia events between the two groups. A total of 267 routine stool examinations were performed, with positive faecal occult blood in 37.45% (100/267) and positive faecal white blood cells in 8.2% (22/267) of patients. There were significant differences between the two groups (*P* < 0.001) (Table 1).

**Table 1:** Clinical characteristics of infants with or without *Clostridium perfringens *colonization

	Positive for* Clostridium perfringens*	Negative for* Clostridium perfringens*	*P* value
Number	112	158	
Male/Female	59/53	92/66	0.3655
Age (mean ± SD) (Months)	2.89±1.85	2.70±3.39	0.5922
Outpatient/inpatient	78/34	90/68	0.0342
Newborns/1-6 months	23/89	53/105	0.0192
Main symptoms and diagnosis			
Bloating	9 (8.04%)	4 (2.53%)	0.0375
Diarrhoea	36 (32.14%)	59 (37.34%)	0.3799
Mucoid and/or bloody stool	63 (56.25%)	39 (24.68%)	<0.0001
NEC*	8 (7.14%)	2 (1.27%)	0.0188
FPIAP*	36 (32.14%)	13 (8.23%)	<0.0001
Colitis	28 (25.0%)	52 (32.91%)	0.1415
Diarrhoea	31 (27.68%)	48 (30.38%)	0.6308
Disease duration			
<2 Weeks	46 (41.07%)	105 (66.46%)	0.0002
2 Weeks to 2 months	48 (42.86%)	39 (24.68%)	
>2 Months	18 (16.07%)	14 (8.86%)	
Feeding patterns			
Exclusive breast feeding	36 (32.14%)	14 (8.86%)	0.0432
Cow’s milk-based formula	24 (21.43%)	102 (64.56%)	
Mixed feeding	52 (46.43%)	42 (26.58%)	
Blood test (n=231)			
White blood cells >12.0×10^9^/L	16 (14.29%)	47 (29.75%)	0.6974
Platelet >400×10^9^/L	39 (34.82%)	3 (1.89%)	<0.0001
Haemoglobin			
>120 g/L	25 (22.32%)	108 (68.34%)	<0.0001
90-120 g/L	53 (47.32%)	39 (24.68%)	
< 90 g/L	5 (4.46%)	1 (0.63%)	
Stool test (n=267)			
Occult blood	64 (57.14%)	36 (22.78%)	0.0019
White blood cell	17 (15.18%)	5 (3.16%)	0.0090
Antibiotics (Metronidazole)	39 (33.9%)	17 (10.76%)	<0.0001

*NEC Necrotizing enterocolitis, *FPIAP food protein-induced allergic proctocolitis

### Clostridium perfringens potential specific toxin genes

Potential specific toxin genes were detected in 85 faecal samples from 30 patients at different time points (0, 2, 4, 12 and 24 weeks). The occurrence rates of *Clostridium perfringens* isolates carrying potential specific toxin genes were as follows: alpha toxin, 97.6% (83/85); beta-2 toxin, 82.3% (70/85); beta toxin, 65.9% (56/85); and no isolates carrying other toxins. Genotypes of 83 samples were identified, of which 67.5% (56/83) were genotype C and 32.5% (27/85) were genotype A. Both genotypes A and genotypes C were detected in 17 patients (Table 1 and Additional file 1: Table S1).

### Food allergy development

**Fig. 2:**
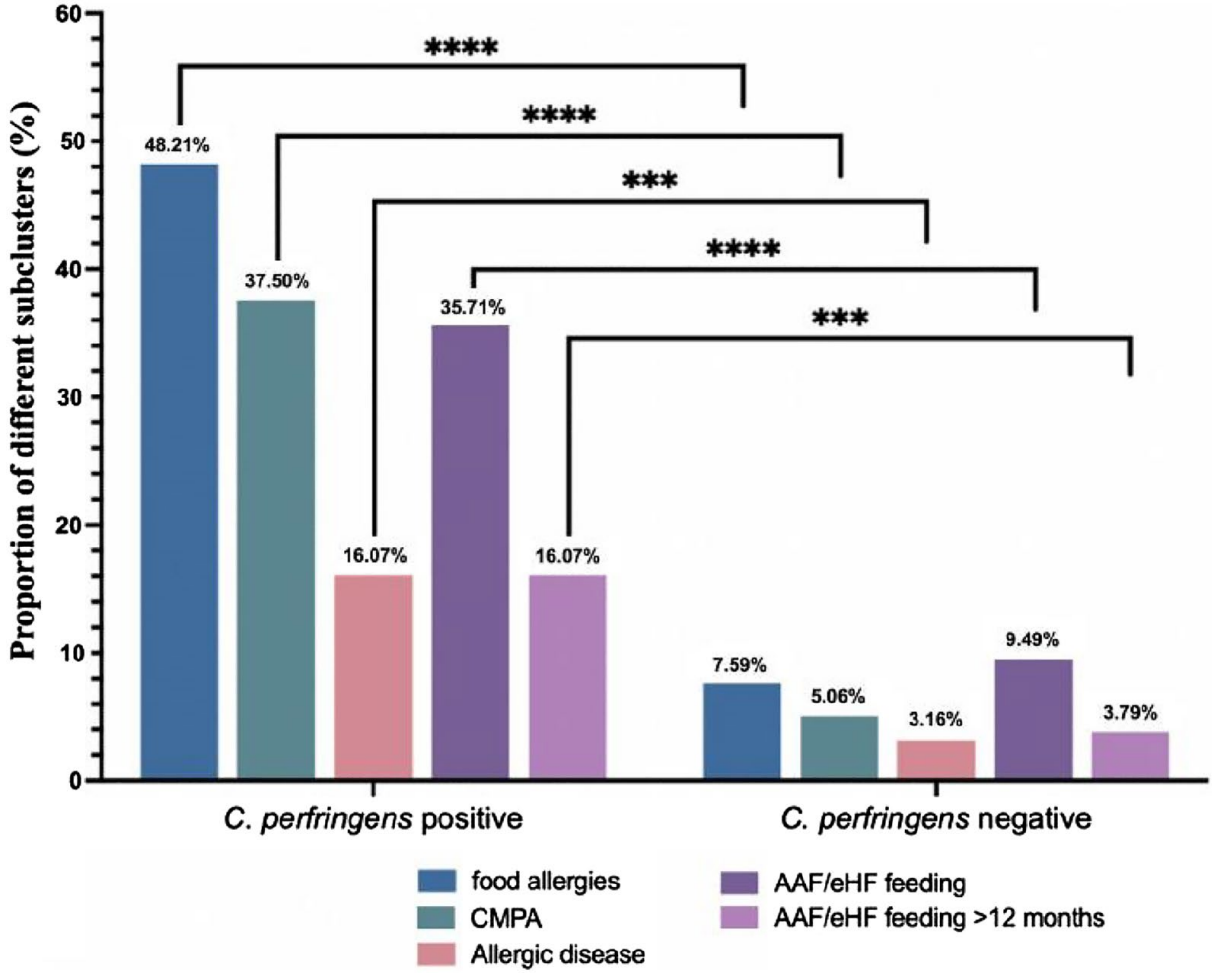
The percentage of food allergies and the use of hypoallergenic formula in the two groups. Bar charts show the proportion of different subclusters in the different groups (*** p < 0.001, **** p < 0.0001)

After a 12-month follow-up survey, 24.44% of cases (66/270) were clinically diagnosed with food allergies based on symptoms, dietary exclusion and oral food challenge, including 18.5% of children (50/270) with CMPA (Fig. 1). In our cohort of *the Clostridium perfringens-*positive group, 48.21% (54/112) developed food allergies, and 37.5% (42/112) developed CMPA, both of which were significantly higher than those in the *Clostridium perfringens-*negative group (7.59% (12/158) and 5.06% (8/158), respectively) (*P* < 0.0001) (Fig. 2). A total of 8.52% (23/270) of the children were diagnosed with allergic diseases, of whom 11 were diagnosed with food allergic gastroenteritis and 12 had eczema and allergic rhinitis: 16.07% (18/112) in the *Clostridium perfringens-*positive group and 3.16% (5/158) in the *Clostridium perfringens*-negative group (P = 0.0002). A total of 20.37% of children (55/270) were fed with amino acid formula (AAF) or extensively hydrolysed formula (eHF), of which 8.89% (24/270) were fed for more than 12 months. The utilization rate of AAF/eHF in the *Clostridium perfringens-*positive group was 35.71% (40/112), which was higher than that in the negative group (9.49%, 15/158) (P < 0.0001). The number of children in both groups who were fed AAF and/or eHF for more than 12 months was 16.07% (18/112) and 3.79% (6/158), respectively (P = 0.0005) (Fig. 2).

### Correlation of Clostridium perfringens colonization and food allergy

**Fig. 3:**
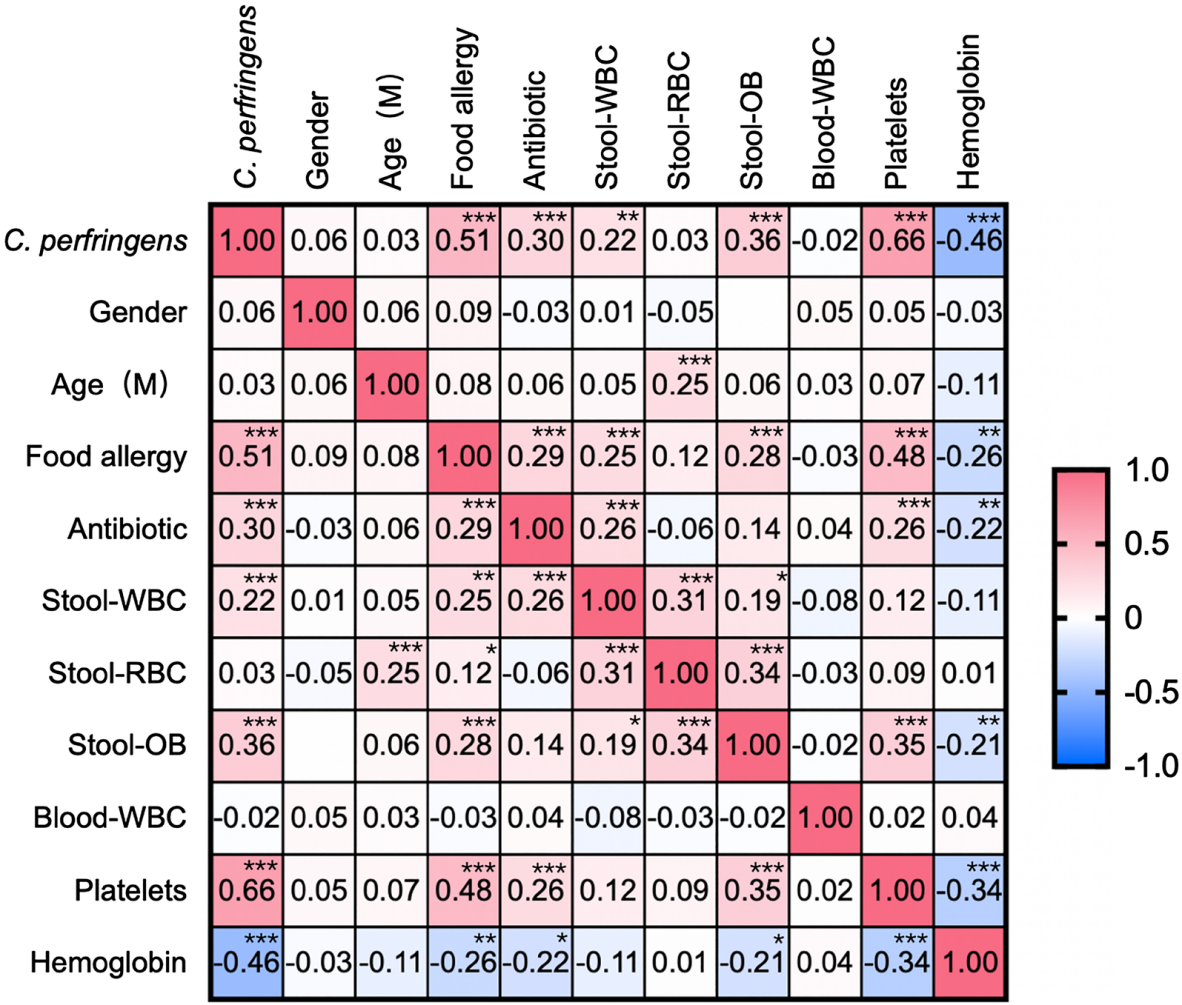
Heatmap showing the correlation characteristics of *Clostridium perfringens *colonization; *P < 0.05; **P < 0.001; ***P < 0.0001

To explore the correlation between *Clostridium perfringens* positivity and food allergy, Pearson correlation analysis was performed, generating a heatmap showing that food allergy was significantly correlated with positivity for *Clostridium perfringens*, as was stool occult blood, white blood cells in the stool, antibiotic use, increased platelets and decreased haemoglobin (*P* < 0.0001) (Fig. 3).

To explore the correlation between *Clostridium perfringens* positivity and food allergy, the heatmap was performed and showed the correlation between food allergies and *Clostridium perfringens* positivity, stool occult blood and white blood cells in stool, antibiotic (metronidazole) use, increased platelets and decreased haemoglobin (Fig. 3).

Platelets have been reported to be involved in food anaphylaxis and are correlated with the severity of anaphylaxis. Our study showed a positive correlation between food allergy and platelets (P < 0.0001), which is consistent with reported studies. The common symptoms of food allergy in infants include diarrhoea and bloody stools, which are often misdiagnosed as acute or chronic enteritis and treated with antibiotics. Persistent blood in the stool may lead to anaemia, which can explain the correlation between food allergy and antibiotic use (P < 0.0001) and haemoglobin level (P = 0.0001) in this study (Fig. 3).

## Discussion

In this real-world evidence study, we attempted to assess the *Clostridium perfringens* colonization rate in Chinese infants with gastrointestinal symptoms and its association with food allergy and CMPA development. Our results showed that 35.75% of infants aged ≤ 6 months with gastrointestinal symptoms had *Clostridium perfringens* colonization. The earliest *Clostridium perfringens* colonization was detected within 2 days after birth. At the 12-month follow-up survey, 24.44% of the patients were clinically diagnosed with food allergies, but the prevalence in the *Clostridium perfringens-*positive group (48.21%) was significantly higher than that in the negative group (7.59%) and was also significantly higher than the reported 11.1% prevalence of food allergies in the general paediatric population [10]. More specifically, we found that 18.52% developed CMPA overall, but the prevalence in the *Clostridium perfringens-*positive group (37.5%) was significantly higher than that in the negative group (5.06%) and was also significantly higher than the 2.69% occurrence reported in our previous study in a general paediatric population [11]. Our results indicated for the first time that *Clostridium perfringens* colonization is high in Chinese infants with gastrointestinal symptoms and is associated with the development of food allergies and CMPA.

*Clostridium perfringens* is widely present in the gastrointestinal tract of healthy humans and animals [12]. Nagpal et al. [7] reported that enterotoxigenic *Clostridium perfringens* remained undetected on day 7 but was detected in 1.1%, 4.5%, 10.1% and 4.5% of infants at 1 month, 3 months, 6 months and 3 years, respectively. Shaw et al. [13] reported that 29.4% of preterm infants were colonized with toxin gene-carrying *Clostridium perfringens* 3 weeks after birth, and they found that maternal breast milk, oxygen administration and antibiotic treatment were inversely associated with the colonization rate. Our study showed that 33.52% of infants with gastrointestinal symptoms had *Clostridium perfringens* colonization, and the rates of *Clostridium perfringens* positivity in neonates and infants aged 1–6 months were 30.25% and 45.88%, respectively. The different colonization rates may have been due to the different patient populations, modes of delivery, feeding methods and gastrointestinal disease statuses.

*Clostridium perfringens* and *Clostridium difficile* are pathogenic clostridia potentially associated with gastrointestinal infections and allergy in infants [8]. In the 1980s, studies reported the testing of sensitization and oral tolerance to ovalbumin and chemicals with *Clostridium perfringens* toxins [14, 15], but the relationship between intestinal colonization by *Clostridium perfringens* and food allergies has not been previously reported. Prior studies have demonstrated that *Clostridium perfringens* colonization may cause a wide variety of pathological conditions ranging from asymptomatic infections to severe life-threatening septic shock, such as gas gangrene, food poisoning, necrotic enteritis, antibiotic-associated diarrhoea, bacteraemia, enterotoxaemia and severe intravascular haemolysis [16–21]. Studies have focused on *Clostridium perfringens* infection and its capacity to generate an array of lethal toxins (seven major toxinotypes A–G) and enzymes, such as lecithinase, fibrinase, hyaluronidase, collagenase and DNA enzymes, which contribute to its invasiveness. Although most identified diarrhoea-associated pathogens were viruses, no specific pathogen was found in almost 80% of reported cases. Possible aetiologies for those cases may include gastrointestinal infections with *Clostridium perfringens* and dietary/environmental factors [22, 23]. There has been no report on whether *Clostridium perfringens* colonization affects the development of food allergies in children. In our cohort of 112 infants with confirmed *Clostridium perfringens* colonization, 48.21% of them developed food allergies, and 37.5% developed CMPA, and both of these prevalences are significantly higher than those in the *Clostridium perfringens-*negative group (7.59% and 5.06%, respectively) and significantly higher than those reported in the general paediatric population. Furthermore, correlation analysis showed *that* food allergy was significantly associated with *Clostridium perfringens* colonization, as well as antibiotic use, stool occult blood, white blood cells in stool, platelets and haemoglobin levels in our cohort. Our results clearly demonstrated that *Clostridium perfringens* colonization in the 1st year of life may promote the development of food allergies and CMPA.

The immunopathological mechanisms by which *Clostridium perfringens* colonization promotes food allergies are still unclear. Research has suggested that *Clostridium perfringens* enterotoxin (CPE) plays a key role in promoting tight junction disassembly and inducing epithelial damage [24], which lead to changes in microbiota and trigger host immune responses [25]. Variations in immune cell populations are largely driven by the environment and microbial antigens [26]. It is interesting that subjects who showed high colonization by Clostridium did not carry *Bacteroides* and *Klebsiella*, both of which were significantly correlated with allergy development [27]. There are only a few studies on *Clostridium perfringens* colonization and food allergy, but the results are inconsistent. Nakayama J reported that allergic infants had high colonization by *Bacteroides* and/or *Klebsiella* and less colonization by *Clostridium perfringens*/butyricum [27]. In contrast, another study reported that Clostridium was more abundant in allergic infants [28, 29]. Roessler et al. [30] proved that the faecal *Clostridium perfringens* cluster I-II levels remained unaffected, suggesting either a change in their activity or the fact that other bacterial species are responsible for the reduced genotoxic activity of faecal water. In summary, we speculate that *Clostridium perfringens* colonization causes invasive gastrointestinal infection due to its enterotoxins, leading to tight junction disassembly, epithelial damage, changes in microbiota, an abnormal host immune response and, eventually, the development of food allergies. Further studies are needed to clarify the underlying immunological and molecular mechanisms of food allergy development in infants colonized with *Clostridium perfringens.*

## Conclusions

We were able to follow a cohort of 270 infants with/without confirmed *Clostridium perfringens* colonization and found that 48.21% of children with *Clostridium perfringens* colonization developed food allergies and 37.5% developed CMPA. Our results demonstrate that *Clostridium perfringens* colonization is associated with the development of food allergies and CMPA in Chinese infants.

## Methods

A real-world case‒control study was carried out at Guangzhou Women and Children’s Medical Center, a tertiary children’s hospital in Guangzhou, China. We included all infants 0–6 months of age who were hospitalized or in outpatient clinics due to gastrointestinal symptoms between January 1st, 2020, and December 31st, 2021. Faecal samples were cultured for detection of *Clostridium perfringens*. Patients’ clinical features, feeding patterns, laboratory tests, and treatment outcomes were documented. Patients with documented congenital metabolic diseases, immune deficiency diseases, and malignant tumours were excluded. A 12-month follow-up interview was conducted, specifically for any clinical diagnosis of food allergies and CMPA, which included eczema, allergic rhinitis, and related conditions such as food protein-induced allergic proctocolitis (FPIAP). Food allergies were clinically diagnosed based on symptoms, dietary exclusion and oral food challenge. The institutional ethics committee of Guangzhou Women and Children’s Medical Center approved this study protocol (202,044,501).

Faecal samples were cultured for the detection of *Clostridium perfringens* and confirmed by flight mass spectrometry. The rectal swab was placed into nutrient broth and delivered to the clinical microbiology laboratory within 2 h of collection; 1 mL of the sample was inoculated into cooked beef medium covered with a layer of sterile liquid paraffin oil to prevent the entry of oxygen into the medium. Then, the medium was heated at 80 °C for 15 min to eliminate competitive bacterial organisms before being cultured at 37 ℃ for 48 h. If much gas was present in the medium due to turbulent fermentation, 15 µL of the broth was transferred to a 5% sheep blood agar plate and streaked from the inoculated area before being cultured anaerobically at 37 ℃ for 24 h. The suspected colonies were identified by matrix-assisted laser desorption/ionization time-of-flight mass spectrometry (MALDI-TOF-MS) (Bruker Daltonics GmbH, Billerica, MA, USA). Isolates were maintained in cooked-meat medium with glycerol (30%) and liquid paraffin oil at − 80 ℃ for long-term storage.

Multiplex PCR was used to detect toxin typing of *Clostridium perfringens* isolates, including α-toxin, β-toxin, ε-toxin and ι-toxin, CPE, NetB, and β2-toxin(Additional file 1: Table S1). The specific primers used for detecting the toxic genes were the same as those described by Rood et al. and Harrison et al. [31, 32]. The PCR conditions were as follows: predenaturation at 94 ℃ for 5 min, followed by high-temperature denaturation for 30 s, annealing at 50 ℃ for 30 s, 90 s at 72 ℃ for 32 cycles, and final extension for 5 min. Then, 1.5% agarose gel electrophoresis was used to examine the PCR products, which were subjected to UV gel imaging. The size of the PCR product was determined according to the marker position.

### Statistical analysis

The present analyses were performed using Microsoft Excel and Graph Pad Prism (v9.0.0). Statistical comparisons were conducted by the chi-squared test and one-way analysis of variance (ANOVA). Data are presented as the mean ± SD for continuous variables and as percentages for categorical variables. A correlation matrix was used to assess the relationship among *Clostridium perfringens* colonization, sex, age, food allergy, stool WBC, stool RBC, stool OB, blood WBC, platelets, and haemoglobin. Significance was set at a *P* value of < 0.05.

### Supplementary information

**Additional file 1: Table S1.** Clostridium perfringens potential specific toxin genes.

## Data Availability

The datasets used and/or analysed during the current study are available from the corresponding author upon reasonable request.

## Abbreviations

C. perfringens: * Clostridium perfringens*
CMPA: Cow’s milk protein allergy
FPIAP: Food protein-induced allergic proctocolitis
NEC: Necrotizing enterocolitis
AAF: Amino acid formula
eHF: Extensively hydrolysed formula
