# Supplementary material for: Molecular characterization of Clostridium perfringens isolates from a tertiary children’s hospital in Guangzhou, China, establishing an association between bacterial colonization and food allergies in infants
Source: Gut Pathog. 2023 Oct 8;15:47. doi: 10.1186/s13099-023-00572-x (PMC10561448; doi:10.1186/s13099-023-00572-x)
Supplement: Supplementary file 1 — Additional file 1: Table S1. Clostridium perfringens potential specific toxin genes. [file 13099_2023_572_MOESM1_ESM.docx]

Table S1. Clostridium perfringens potential specific toxin genes

| Sample number | a-toxin | b-toxin | i-toxin | e-toxin | CPE | NetB | Genotypes | b2-toxin |
| --- | --- | --- | --- | --- | --- | --- | --- | --- |
| CP-001-0W | + | - | - | - | - | - | A | + |
| CP-001-12W | + | + | - | - | - | - | C | + |
| CP-002-0W | + | + | - | - | - | - | C | + |
| CP-002-2W | + | + | - | - | - | - | C | + |
| CP-002-4W | + | + | - | - | - | - | C | + |
| CP-003-0W | + | + | - | - | - | - | C | + |
| CP-003-12W | + | + | - | - | - | - | C | + |
| CP-003-16W | + | - | - | - | - | - | A | + |
| CP-003-2W | + | + | - | - | - | - | C | + |
| CP-003-4W | + | + | - | - | - | - | C | + |
| CP-004-0W | + | - | - | - | - | - | A | + |
| CP-004-12W | - | - | - | - | - | - | - | - |
| CP-004-24W | + | - | - | - | - | - | A | + |
| CP-004-2W | + | - | - | - | - | - | A | + |
| CP-004-4W | + | + | - | - | - | - | C | + |
| CP-005-0W | + | + | - | - | - | - | C | + |
| CP-005-12W | + | + | - | - | - | - | C | - |
| CP-005-2W | - | - | - | - | - | - | - | - |
| CP-005-4W | + | + | - | - | - | - | C | - |
| CP-006-0W | + | + | - | - | - | - | C | + |
| CP-006-24W | + | + | - | - | - | - | C | + |
| CP-007-0W | + | + | - | - | - | - | C | + |
| CP-007-12W | + | + | - | - | - | - | C | + |
| CP-007-24W | + | - | - | - | - | - | A | + |
| CP-007-4W | + | + | - | - | - | - | C | + |
| CP-008-0W | + | + | - | - | - | - | C | + |
| CP-009-0W | + | + | - | - | - | - | C | + |
| CP-009-2W | + | - | - | - | - | - | A | + |
| CP-009-4W | + | + | - | - | - | - | C | - |
| CP-010-0W | + | + | - | - | - | - | C | + |
| CP-010-24W | + | + | - | - | - | - | C | - |
| CP-010-2W | + | + | - | - | - | - | C | + |
| CP-010-4W | + | + | - | - | - | - | C | + |
| CP-011-0W | + | + | - | - | - | - | C | + |
| CP-011-12W | + | - | - | - | - | - | A | - |
| CP-011-24W | + | - | - | - | - | - | A | - |
| CP-012-12W | + | + | - | - | - | - | C | + |
| CP-012-24W | + | - | - | - | - | - | A | + |
| CP-012-2W | + | + | - | - | - | - | C | + |
| CP-012-4W | + | + | - | - | - | - | C | + |
| CP-013-4W | + | - | - | - | - | - | A | + |
| CP-014-0W | + | - | - | - | - | - | A | + |
| CP-014-10W | + | + | - | - | - | - | C | + |
| CP-014-12W | + | + | - | - | - | - | C | - |
| CP-014-24W | + | + | - | - | - | - | C | + |
| CP-014-4W | + | + | - | - | - | - | C | + |
| CP-015-0W | + | - | - | - | - | - | A | + |
| CP-015-12W | + | + | - | - | - | - | C | + |
| CP-015-24W | + | - | - | - | - | - | A | - |
| CP-015-2W | + | + | - | - | - | - | C | + |
| CP-016-12W | + | + | - | - | - | - | C | + |
| CP-016-24W | + | - | - | - | + | - | A | + |
| CP-016-4W | + | - | - | - | - | - | A | + |
| CP-017-12W | + | + | - | - | - | - | C | + |
| CP-017-24W | + | + | - | - | - | - | C | + |
| CP-019-12w | + | + | - | - | - | - | C | + |
| CP-019-24w | + | + | - | - | - | - | C | + |
| CP-019-2w | + | - | - | - | - | - | A | + |
| CP-019-4w | + | + | - | - | - | - | C | + |
| CP-020-12w | + | + | - | - | - | - | C | + |
| CP-020-24w | + | - | - | - | - | - | A | + |
| CP-021-12W | + | + | - | - | - | - | C | + |
| CP-021-24W | + | + | - | - | - | - | C | + |
| CP-021-2W | + | - | - | - | - | - | A | + |
| CP-021-4W | + | + | - | - | - | - | C | + |
| CP-022-12W | + | + | - | - | - | - | C | + |
| CP-022-24W | + | - | - | - | - | - | A | - |
| CP-022-4W | + | - | - | - | - | - | A | + |
| CP-023-24W | + | - | - | - | - | - | A | - |
| CP-023-4W | + | - | - | - | - | - | A | + |
| CP-024-12W | + | + | - | - | - | - | C | + |
| CP-024-24W | + | + | - | - | - | - | C | + |
| CP-024-2W | + | - | - | - | - | - | A | - |
| CP-024-4W | + | + | - | - | - | - | C | + |
| CP-025-12W | + | + | - | - | - | - | C | + |
| CP-025-2W | + | - | - | - | - | - | A | + |
| CP-025-4W | + | - | - | - | - | - | A | - |
| CP-026-12W | + | - | - | - | - | - | A | + |
| CP-026-24W | + | + | - | - | - | - | C | + |
| CP-026-2W | + | + | - | - | - | - | C | + |
| CP-026-4W | + | + | - | - | - | - | C | + |
| CP-027-0W | + | + | - | - | - | - | C | + |
| CP-028-0W | + | + | - | - | - | - | C | + |
| CP-029-0W | + | + | - | - | - | - | C | - |
| CP-030-0W | + | + | - | - | - | - | C | + |
